# 395. Impact of Racial Disparities on *Clostridioides difficile* Infection (CDI) Outcomes at a Southern California Academic Teaching Hospital

**DOI:** 10.1093/ofid/ofac492.473

**Published:** 2022-12-15

**Authors:** Anna Y Zhou, Jina M Lee, Natalie M Ortiz-Gratacos, Pharmacy Student, Almas Al Isso, Karen K Tan, Jacinda C Abdul-Mutakabbir

**Affiliations:** Loma Linda University Medical Center, RANCHO CUCAMONGA, California; Loma Linda University Medical Center, RANCHO CUCAMONGA, California; Loma Linda University, Loma Linda, California; Loma Linda University School of Pharmacy, la mesa, California; Loma Linda University Medical Center, RANCHO CUCAMONGA, California; Loma Linda University, Loma Linda, California

## Abstract

**Background:**

Infectious diseases are the second leading contributor to racial disparities in healthcare. *Clostridioides difficile* infection (CDI) is a common community/hospital acquired infection that can cause life-threatening colitis. Despite the influence that socioeconomic factors, insurance status, and access to care can have on CDI outcomes, there is a dearth of literature that evaluates the impact of racial differences. The objective of this study was to identify racial disparities in CDI and describe clinical outcomes among hospitalized adults.

**Methods:**

This is an IRB approved, retrospective study of adult patients (≥ 18 years) hospitalized with initial episodes of CDI between January 2020 to June 2021. Initial CDI episodes were identified by the ICD-10 code A04.72. Medical records were reviewed for demographics, comorbid conditions, insurance, total length of stay (LOS), and need for intensive care unit (ICU) admission. Patients were dichotomized by race/ethnicity into either the racially and ethnically minoritized (REM) group or the non-Hispanic White (n-REM) group. P value of ≤ 0.05 was defined as significant.

**Results:**

In total 220 patients (61.8% REM, 136/220; 38.2% n-REM, 84/220) with an initial CDI episode were included. Majority of the REM patients were Hispanic (38.2%) followed by Black (13.6%), and Asian (5%). Compared to the n-REM group, the REM patients were younger (60 vs 69 years, p =0.016) and were more likely to have underlying comorbidities (diabetes mellitus 33% vs 9.5%; p < 0.001 and chronic kidney disease 48.5% vs 21.3%; p < 0.001). The REM patients were more likely to present with fulminant CDI (21.3% vs 11.9%; p =0.1). The REM patients were also more likely to be admitted to the ICU during their stay (41.9% vs 26.2%; p =0.021) and have a longer hospital length of stay (LOS) (12 vs 7 days; p =0.02). Lastly, the REM patients were more likely to be under-insured with 91 patients (66.9%) having Medi-Cal insurance compared to 34 patients (40.5%) in the n-REM group (p < 0.001).

**Conclusion:**

Despite being younger, REM patients were more likely to require an ICU admission, have underlying comorbidities, have a longer LOS, and be under-insured. There is a need for further studies to understand disparities between REM and n-REM patients regarding CDI.

**Disclosures:**

**All Authors**: No reported disclosures.

